# Cobas Ampliprep/Cobas TaqMan HIV-1 v2.0 Assay: Consequences at the Cohort Level

**DOI:** 10.1371/journal.pone.0074024

**Published:** 2013-08-30

**Authors:** Ninon Taylor, Katharina Grabmeier-Pfistershammer, Alexander Egle, Richard Greil, Armin Rieger, Bruno Ledergerber, Hannes Oberkofler

**Affiliations:** 1 Department of Internal Medicine III, Paracelsus Medical University, Salzburg, Austria; 2 Department of Dermatology, Vienna Medical University, Vienna, Austria; 3 Division of Infectious Diseases and Hospital Epidemiology, University Hospital Zürich, University of Zürich, Zürich, Switzerland; 4 Department of Laboratory Medicine, Paracelsus Medical University, Salzburg, Austria; Rush University, United States of America

## Abstract

**Background:**

High-sensitive real-time PCR assays are routinely used to monitor HIV-1 infected subjects. Inter-assay discrepancies have been described at the low viral load (VL) end, where clinical decisions regarding possible virological rebound are based.

**Methods:**

A retrospective study was performed to analyze frequencies of viral blips after transition to the COBAS Ampliprep/COBAS TaqMan v2.0 HIV-1 assay (Taqman v2.0) in patients with prior undetectable VLs as measured with the Roche Cobas Ampliprep Amplicor HIV-1 Monitor Test, v1.5 (Amplicor) and was evaluated in comparison to a group of patients monitored with the Abbott Real-time HIV-1 assay (Abbott RT) during the same period of time.

**Results:**

85 of 373 patients with VLs below the limit of quantification with Amplicor had VLs >50 copies/mL after transition to the TaqMan v2.0 assay. Among these 74.1% had VLs ranging from 50–499 copies/mL, 22.9% had VLs >500 copies/mL. From 22 patients with initial Taqman v2.0 based VLs exceeding 500 copies/mL, 6 patients had VLs <20 copies/mL after novel VL measurement on a next visit. In our control group with VL quantification using the Abbott RT assay, only 1 patient became detectable and showed a VL of <40 copies/mL after new measurement.

**Conclusions:**

Transition to the Taqman v2.0 assay was accompanied by an increase of quantifiable HIV-1 VLs in patients with long term viral suppression under antiretroviral therapy that might be attributed to technical shortcomings of the Taqman v2.0 assay. A high test variability at the low VL end but also beyond was observed, making meaningful clinical interpretation of viral blips derived from different assays difficult.

## Introduction

Viral load (VL) quantification constitutes a fundamental cornerstone of antiretroviral therapy management in HIV-1 infected subjects [1]. New, ultrasensitive real-time (RT) -PCR assays for HIV-1 RNA quantification have been developed during the last years including the Abbott Real Time HIV-1 assay (Abbott RT, Abbott Diagnostics, Wiesbaden Germany) and the COBAS Ampliprep/COBAS TaqMan HIV-1 assay (TaqMan, Roche Diagnostics, Mannheim, Germany). Both test systems differ significantly regarding their extraction system, primer target region and probe design.

Shortly after the first version of the TaqMan assay (TaqMan v1.0), which targets the *gag* region, was released, numerous studies reported unusual findings including false negative or underestimated VL measurements in patients monitored with the TaqMan v1.0 assay [2]–[6]. Single point mutations in the downstream primer of the TaqMan v1.0 assay were identified as a leading cause for these observations [5], resulting in the development of an upgraded TaqMan v2.0 assay using multiplex amplification and fluorescence detection of two distinct genomic regions, the long terminal repeat and gag region, respectively. Furthermore, the limit of quantification (LOQ), as defined by the manufacturer, was reduced from 40 copies/mL to 20 copies/mL. We have shown previously that the TaqMan v2.0 and the Abbott RT assay both allowed accurate determination of viral load levels in individuals infected with HIV-1 isolates that were found falsely negative or underestimated with the Roche CTM v1.0 assay [7].

Although the overall reliability and accuracy of the TaqMan v2.0 and the Abbott RT assay, targeting the integrase region, were found to be similar [7]–[9], initial reports describe a significant variability between these assays around the LOQ [10]. We therefore compared frequencies of viral blips in two Austrian HIV-1 patients care centers monitoring HIV-1 VL with the Taqman v2.0 or the Abbott RT assay, respectively.

## Methods

Plasma samples were recruited from HIV-1 infected individuals from two Austrian HIV outpatient clinics in Salzburg (Salzburg Center), and in Vienna (Vienna Center). Both HIV centers are part of the Austrian HIV Cohort (AHIVCOS) and the study has been approved by the ethics committee of the Vienna Medical University (No. 898/2010) and the ethics committee of the Salzburg Federal Government (No. 1159/2010), respectively. Written informed consent was given by the patients for their information to be stored in the hospital database and used for research.

Both centers followed similar hospital procedures for routine venipuncture and blood collection in ethylene diamine tetra acetic acid (EDTA) anticoagulated tubes. Blood was collected in 10 mL BD Vacutainer EDTA tubes and transferred within 2–4 h post collection from the clinic site to the laboratory for further processing. The EDTA tubes were centrifuged at arrival in the diagnostic laboratory for 10 min at 1,100 g for plasma separation. After centrifugation, 1 mL of plasma was transferred into secondary tubes and stored at −20°C. On the day of analysis, the plasma aliquots were thawed, vortexed and analyzed for viral load.

We performed a retrospective analysis comparing both centers in order to evaluate whether significant differences in the number of elevated VL measurements in formerly virologically suppressed patients after implementation of the TaqMan v2.0 assay could be found.

On August 6, 2009 the Vienna Center switched from the Cobas Ampliprep/ Amplicor HIV-1 Monitor Test v1.5 (Amplicor) (Roche Diagnostics, Mannheim, Germany) to real-time PCR technology using TaqMan v2.0. Throughout the same period of time, the Salzburg Center used Abbott RT testing for HIV-1 quantification, which had formerly been introduced in August 2008. Inclusion criteria were chosen as follows: 1) Initiation of ART prior to August 6, 2008, 2) ≥1 VL measurement in the pre TaqMan v2.0 period from August 6, 2008 to August 5, 2009 (designated as time point 1) and all VL measurements below the LOQ (Amplicor: <50 copies/mL, Abbott RT: <40 copies/mL) and no ART changes; 3) ≥1 VL measurement during the TaqMan v2.0 period from August 6, 2009 to August 5, 2010 (designated as time point 2) and no ART changes. Furthermore, patients who reached VLs above 20 copies/mL at time point 2, were reassessed with a new VL measurement on their next clinical visit (designated as time point 3). For the Amplicor and the TaqMan v2.0 assay, automated RNA extraction was performed using the COBAS Ampliprep system. The Abbott m24 sp automated sample preparation system was used for RNA isolation for the Abbott RT HIV-1 assay. PCR amplification was then performed either on the COBAS Amplicor system using the Amplicor assay with a dynamic range from 50 to 750.000 copies/mL, on the COBAS TaqMan 48 system using the TaqMan v2.0 assay with a dynamic range from 20 to 10.000.000 copies/mL or on the m2000 rt Real- Time PCR system using the Abbott RT HIV-1 assay with a dynamic range of 40 to 10.000.000 copies/mL. Genotypic drug resistance testing was performed using the ViroSeq® HIV-1 Genotyping System (Abbott Diagnostics).

## Results

Following the switch to the TaqMan v2.0 assay in the Vienna Center, multiple internal reports accumulated concerning an increase of detectable VLs in patients with previous long-term virological suppression.

In the Vienna Center, 373 of 2078 recruited HIV-1 infected individuals met the inclusion criteria. 288 (77.2%) patients remained with a HIV-1 VL below 50 copies/mL after implementation of the TaqMan v2.0 assay. Due to increased dynamic range of the TaqMan v2.0 assay 67 patients now had quantifiable VL levels ranging from 20 to 49 copies/ml and 221 patients had VL measurements <20 copies/ml. Interestingly for 85 subjects (22.8%) VLs >50 copies/mL were reported. Among these patients, 63 had VLs ranging from 50–499 copies/mL while 22 patients showed VLs >500 copies/mL (Table 1). As a consequence all patients with VLs >20 copies/mL as measured by TaqMan v2.0 were reassessed at time point 3. For 20 patients, VL control could not be performed. The 132 novel VL quantifications using again TaqMan v2.0 are shown in table 2. The majority of patients that had a VL between 20 and 499 copies/mL at time point 2 were below 20 copies/mL at time point 3. Remarkably, in the group of patients who when switching to TaqMan v2.0, reached at once VLs exceeding 500 copies/mL, 28.6% became undetectable after novel VL measurement using again TaqMan v2.0, raising questions about potential assay variability at low detection limit but also beyond (Table 2). For three patients, additional genotypic resistance tests were performed in response to the observed viral blips. One resistance test revealed a new M184V mutation, another patient had two new minor protease inhibitor mutations with no consequences for his ongoing therapy. The third genotypic testing originated from a salvage patient with no former resistance testing available, so that no clinical conclusion may be drawn in this setting. Furthermore, 6 patients underwent subsequent ART changes as stated in their case history.

**Table 1:** HIV-1 viral load (VL) measurements after the switch to the Taqman v2.0 assay in patients under stable ART and with VL levels below the limit of quantification (<50 copies/ml) with the Amplicor assay for ≥1 year.

COBAS Ampliprep/COBAS TaqMan v2.0 HIV-1 assay					
VL	<20	20–49	50–499	>500	Total
N (%)	221 (59.2)	67 (18.0)	63 (16.9)	22 (5.9)	373 (100)

VL: HIV-1 viral load levels representing copies /mL; N: number of patients; Amplicor: Roche Cobas Ampliprep Amplicor HIV-1 Monitor Test, v1.5; TaqMan v2.0: COBAS Ampliprep/COBAS TaqMan v2.0 HIV-1 assay.

**Table 2:** Comparison of HIV-1 viral load measurements in patients using the Taqman v2.0 assay at two different time points.

	COBAS Ampliprep/COBAS TaqMan v2.0 HIV-1 assay				
	VL - TP3 <20 N (%)	VL - TP3 = 20 – 49 N (%)	VL - TP3 = 50 – 499 N (%)	VL - TP3 >500 N (%)	n.d.
**VL - TP2 = 20**–**49**	36 (63.2%)	15 (26.3%)	5 (8.8%)	1 (1.7%)	10
**VL - TP2 = 50**–**499**	30 (55.6%)	9 (16.7%)	14(25.9%)	1(1.8%)	9
**VL - TP2 >500**	6 (28.6%)	4 (19.0%)	6 (28.6%)	5 (23.8%)	1

VL-TP2; HIV-1 viral load (copies/ml) at time point 2; VL-TP3; HIV-1 viral load (copies/ml) at time point 3; N: number of patients; n.d.: not determined; TaqMan v2.0: COBAS Ampliprep/COBAS TaqMan v2.0 HIV-1 assay.

During the same time period, 288 HIV-1 infected individuals were recruited in the Salzburg Center and 52 patients met our inclusion criteria. For these patients, VL quantification was performed continuously using the ABB assay and only 1 patient became detectable (137 copies/mL) from August 2008 to August 2010. A new measurement resulted in a VL of <40 copies/mL at the next clinical visit. In addition a direct comparison of the TaqMan v2.0 and the Abbott RT assay was performed at the Salzburg Center using 77 samples originating from HIV-1 infected subjects under ART with previous long-term virological suppression and with HIV-1RNA levels below the LOQ with at least one of the two assays. One sample had a VL of 76 copies/ml as measured with the Abbott RT assay but was undetectable with the TaqMan v2.0 assay whereas 12 had a VL of >50 copies/ml with the TaqMan v2.0 assay but were below the limit of detection with the Abbott RT assay (data not shown).

## Discussion

Transition from the Amplicor assay to the TaqMan v2.0 assay in the Vienna Center was followed by an increase of quantifiable VLs in patients with stable ART and prior successful viral suppression, which at least in part, could not be reproduced in subsequent VL measurements. We are aware that our findings are not derived from repeat measurements of the same samples but from sequential clinical visits.

The implementation of newer high-sensitive RT-PCR assays with lower detection limits for HIV-RNA VL monitoring has been shown to lead to increased frequency of blips [11]–[14]. Over the past years, different explanations were proposed, especially concerning probe handling. For instance, freezing plasma in situ in a plasma preparation tube (PPT) containing a gel barrier for separation of blood cells from plasma upon centrifugation was associated with higher HIV-1 VL results as compared to plasma from standard EDTA tubes when tested with the Amplicor [15], [16] and TaqMan v1.0 [17]. The disparity in quantification was more apparent in specimens with low viremia and was associated to transportation issues after centrifugation in PPT tubes. However, both our centers have been continuously using standard EDTA containing tubes without gel separation, for which increased levels of HIV-1 RNA have not been reported [15], [18]. Further, the most obvious handling failure in clinical settings concerning standard EDTA tubes is postponed centrifugation and plasma transfer more than 6 h after sample collection that could lead to HIV-RNA degradation.

A study by Garrett et al compared the size and rate of blips between TaqMan v1.0 and 2.0 in a cohort of virologically suppressed HIV-1 patients and found similar blip rates for both test systems but a higher amplitude of blips with v2.0. Also a higher proportion of blips on TaqMan v2.0 exceeding a cut-off of 200 copies/mL was observed [19]. Previous studies have also shown that a significant percentage of HIV-1 infected patients receiving ART with undetectable VLs as monitored with the Abbott RT assay, tested positive with the TaqMan v2.0 assay [7], [10], [20] which is in line with our findings in 77 samples with HIV-1RNA levels below the LOQ with at least one of the two assays. A recently published paper by Naeth et al evaluated precision and concordance of both systems using longitudinal specimens from HIV-1 infected patients with stable CD4+ cell counts and found significantly higher mean VL in low viremic samples with TaqMan v2.0 as compared with Abbott RT. Additionally, higher coefficients of variation (37–59% with TaqMan v2.0 vs. 26–31% with Abbott RT) as determined by replicate testing of three specimen samples were calculated using non-log transformed data [10]. This phenomenon may reflect other issues than handling procedures such as undesirable additive effects resulting from the utilization of the same fluorescent dye for labeling of the two TaqMan probes, targeting the *LTR* and *gag* region, respectively. Although we are aware that our study does not provide any mechanistically insight this technical issue might be addressed in the future by using different fluorescent dyes for each target and calculating VLs for each target from separate calibration curves allowing comparison of two VL measurements in the same sample within the same run.

The clinical significance of viral “blips” in previously stable patients is still a matter of debate and remains a source of dilemma for many clinicians. Recently, it was shown that virological blips of >500 copies/mL were associated with increased rebound risk, whereas no significant association was observed for blips between 50–500 copies/mL [21]. However, more recently, an analysis by Geretti et al showed evidence that reliable HIV-1 detection at levels even below 50 copies/mL as determined by Abbott RT is clinically meaningful for treatment efficacy [22]. Furthermore, the exact etiology of “blips” is still unclear, reaching from HIV rebound from reservoirs [23] or ongoing cycles of replication [24], to random statistical fluctuation or methodological issues, especially concerning the high variability of RT-PCR assays at the LOQ [25]. Intermittent VL elevation is also seen due to immune activation after vaccination or in face of concomitant infection such as syphilis. The latest version of the Department of Health and Human Services (DHHS) guidelines as well as the AIDS Clinical Trial Group (ACTG) recommend to define virological rebound as a confirmed VL of >200 copies/mL [26].

In an attempt to address the socio-economic impact of our findings, we noted that after introduction of the TaqMan v2.0 assay in the Vienna Center 132 additional VL repeat measurements as well as 3 genotypic testings were required. Considering 58€ per VL test, 16€ per CD4 cell count, 5€ per venipuncture and 171€ per genotypic resistance test, the overall cost for additional laboratory testing reached 11000€ not including the supplementary financial burden due to ART modification. Our very conservative estimate does also not take into account the increased workload and time required by clinic staff as well as the psychological stress for patients confronted with the possibility of an emerging drug resistance and numerous adherence discussions between patients and physicians.

In summary, we show that transition from the Amplicor assay to a high-sensitive, dual-target RT-PCR assay (TaqMan v2.0) led to an increased frequency of reported quantifiable VLs in patients with long term viral suppression under ART. Although the control group was considerably smaller than our group of interest in Vienna, the comparison with a second center with the same clinical standards of care using the Abbott RT assay for VL monitoring questions the reliability of the Taqman v2.0 assay at the lower VL end. Furthermore, the variability of VL measurements in patients with VL above 500 copies/mL as determined by TaqMan v2.0 was surprising and constitutes an additional matter of concern. For clinicians interpretation of single detectable HIV VLs especially when longitudinal data are derived from different assays is very difficult and should not lead to immediate genotypic testing or therapy intensification.
